# Notes and News

**Published:** 1902-02-01

**Authors:** 


### NOTES AND NEWS.

HOSPITAL MEETINGS.

Queen Charlotte's Hospital.—Annual Meeting at the hospital on Monday, February 24th, at 3 P.M.

Charing Cross Hospital.—Annual Meeting at the hospital on February 19th, at 2 P.M.

Seamen's Hospital, Greenwich.—Annual Meeting at the Institute of Chartered Accountants on February 28th, at 3.30 P.M.

Great Northern Central Hospital.—Annual Meeting on February 28th, at 5 P.M., at the hospital.

The Middlesex Hospital.—Annual Meeting at the hospital on February 27th, at 12 noon.

King's College Hospital.—Annual Meeting at the hospital on February 27th, at 4 P.M.

The Cancer Hospital (Free).—Annual Meeting at the hospital on February 26th, at 4 P.M.

The Metropolitan Hospital Saturday Fund distribution this year amounts to £19,214 3s. 4d., and will be received by 178 institutions.

The actual expenditure upon site, buildings, and equipment of the Grove Hospital of the Metropolitan Asylums Board was £279,567 17s. 11d.

Col. Bowdler presented the South African war medals to the members of the Metropolitan corps of the St. John's Ambulance Brigade on Monday.

The Metropolitan Asylums Board hold that they cannot insist that vaccination should be a condition necessary for visitors to the small-pox hospitals, but they take the precaution of sending the names and addresses of visitors to the medical officer in the district in which they reside. In the case of public libraries which might easily become a source of danger the librarians receive daily notice of all cases of infectious disease, and books coming from infected dwellings are disinfected and in the case of small-pox, they are destroyed.

The late Mr. W. Brocksopp, of Holloway, has bequeathed £200 each to the Royal Free, Great Northern Central, Metropolitan, St. Peter's Hospital for Stone, Small pox Hospital, Holloway, Hospital for Diseases of the Chest, the Ophthalmic Hospitals of Bloomfield Street and Gray's Inn Road, National Hospital for Diseases of the Heart, National Hospital for the Paralysed and Epileptic, New Hospital for Women, and £300 each to the London Hospital, the Cancer Hospital, and the Hospital for Women, Soho Square. Many other hospitals will receive £100 each.

A cot has been endowed at the Royal Hospital, Richmond, by Mrs. Owen, of Sheen Lodge, Richmond Park, in memory of her two sons, grandsons of Sir Richard Owen, K.C.B. The cot is the outcome of a wish of the elder son that his mother should "do something for the good of the hospital."

At the last meeting of the Metropolitan Asylums Board Admiral Adeane, in presenting the report of the Ambulance Committee, said that the Board's new steamer, the *Red Cross*, had arrived in the Thames, and was now ready for work. It would put them in the position, he said, of being able to carry 345 patients per day to the hospital ships.

A severe outbreak of small-pox has occurred at the Mile End Workhouse Infirmary. Thirty-one cases broke out simultaneously. All the patients have been removed and the place disinfected. The origin is not clearly traced. The number of new small-pox cases has shown the greatest variation during the past week, on one day reaching the highest number during the outbreak, and the next day diminishing very greatly.

The Mount Vernon Hospital for Consumption at Hampstead is to be congratulated that it has found not only the way but the means to extend its benefits. The generous and anonymous gift of £100,000 for the establishment of a convalescent branch is most usefully bestowed. The hospital, which is now overwhelmed with applicants, will be able to utilise its forces to far greater advantage when the convalescents can be removed to make way for the cases requiring actual hospital treatment.

The General Purposes Committee of the London School Board have recommended the appointment of Dr. James Kerr as medical officer of the board. Dr. Kerr is medical officer of the Bradford School Board. He has had a distinguished academic and medical career, has held several provincial hospital appointments, and had experience in hygienic supervision of schools. He has made investigations in the outbreak of epidemics, and has examined and reported on school plans. There were 148 candidates for the post.

On Thursday last week (January 23rd) Lord Dudley, the new President of the National Hospital for the Paralysed and Epileptic, superintended the counting of the ballot papers cast on the poll demanded by those governors who desired that at least two members of the old board of management should find seats on the new body. The result was the election of the 10 members nominated by the Committee of Selection. The Rev. Prebendary Henry Wace, D.D., received 243 votes; Mr. Ernest de la Rue, 234; Mr. Frederick Orridge MacMillan, 202; Mr. Edgar Speyer, 202; Sir John Rahere Paget, 198; Mr. Melvill Green, 192; Sir Alexander Mackenzie, 191; Mr. Arnold Royle, C.B., 191; Mr. James Wigan, 191; and Mr. John Danvers Power, 190. The opposition nominees, who failed to secure election, were Mr. John Pearman, for whom 169 votes were cast; and Mr. Frank C. Capel, who received 168. Thus ends an unhappy episode in the history of a famous institution.

